# Engineered myeloid precursors differentiate into osteoclasts and resorb heterotopic ossification in mice

**DOI:** 10.3389/fbioe.2024.1491962

**Published:** 2024-11-22

**Authors:** Cameron Rementer, Apichai Yavirach, Worakanya Buranaphatthana, Philip A. Walczak, Mei Speer, Kat Pierce, Subramanian Dharmarajan, Elizabeth Leber, Bruce Sangiorzan, Steven Bain, Marta Scatena, Alexander Blümke, Cecilia M. Giachelli

**Affiliations:** ^1^ Department of Bioengineering, School of Medicine, University of Washington, Seattle, WA, United States; ^2^ Department of Oral Health Sciences, School of Dentistry, University of Washington, Seattle, WA, United States; ^3^ Department of Prosthodontics, Faculty of Dentistry, Chiang Mai University, Chiang Mai, Thailand; ^4^ Department of Oral Biology and Oral Diagnostic Sciences, Faculty of Dentistry, Chiang Mai University, Chiang Mai, Thailand; ^5^ Department of Orthopedics and Sports Medicine, University of Washington, Seattle, WA, United States; ^6^ Department of Orthopedics and Trauma Surgery, Medical Faculty Mannheim, Heidelberg University, Mannheim, Germany

**Keywords:** bone, heterotopic ossification, osteoclasts, engineered osteoclasts, RANK, chemical inducer of dimerization, resorption

## Abstract

**Introduction:**

Heterotopic ossification (HO) occurs following orthopedic trauma, spinal cord injuries, brain trauma and limb amputations. Once symptomatic, HO causes pain, limited mobility and decreased quality of life. Current treatments are limited and have significant complications with high recurrence rates, underscoring the need for improved therapeutic interventions. Osteoclasts (OCs) are physiological bone resorptive cells that secrete enzymes and protons to degrade bone.

**Methods:**

In this study, we describe the use of genetically engineered OCs as a novel cell therapy approach to treat HO. Inducible, engineered myeloid precursors (iRANK cells) treated with a chemical inducer of dimerization (CID) differentiated into TRAP^+^ multinucleated OCs and resorbed mineralized tissues *in vitro*.

**Results:**

*In vivo*, BMP-2-induced murine HO lesions were significantly regressed following treatment using iRANK cells with concomitant systemic administration of CID. Moreover, many OCs were TRAP^+^, MMP9^+^, and GFP^+^, indicating that they differentiated from delivered iRANK cells.

**Discussion:**

In summary, these data con rm the ability of engineered myeloid precursors to differentiate into OCs and resorb HO *in vivo* paving the way for OC delivery as a promising approach for HO treatment.

## 1 Introduction

Heterotopic ossification (HO) is the abnormal formation of extraskeletal bone and is associated with genetic diseases (hereditary) such as fibrodysplasia ossificans progressiva (FOP) and Albright’s hereditary osteodystrophy (AHO) (Sun et al., 2022; Zheng et al., 2024) or can occur following orthopedic trauma, spinal cord injuries, brain trauma, limb amputations and surgical interventions (acquired) (Bossche and Vanderstraeten, 2005; Łȩgosz et al., 2019; Franz et al., 2022). Acquired HO is the more common form and is currently thought to result from an enhanced osteogenic cytokine and progenitor cell milieu present near sites of injury (Davies et al., 2018; Cappato et al., 2020). It is a growing problem, especially for veterans injured in combat (Xu et al., 2022). One study revealed that up to 82% of soldiers developed HO if they had a spinal cord injury after being wounded by an improvised explosive device (Melcer et al., 2011). In addition, HO is a frequent complication of amputation, joint arthroplasty and orthopedic surgeries involving the hip and elbow (Cappato et al., 2020). HO is often asymptomatic; however, depending on size and location, it can cause pain, ankylosis, immobility, and decreased quality of life (Bossche and Vanderstraeten, 2005; Cappato et al., 2020).

There are currently several approaches that have been approved to prevent and treat HO. Nonsteroidal anti-inflammatory drugs, radiation therapy, or a combination of the two, have been used to manage inflammation and chronic pain, with limited success in the prophylaxis of HO (Xu et al., 2018; Kazezian and Bull, 2021). At advanced stages, surgical excision of the ectopic bone is often the only effective intervention (Kazezian and Bull, 2021). However, surgical excision often results in perioperative complications such as neurovascular injuries, hemorrhage, and the recurrence of HOs, which may increase in size (Xu et al., 2018; Kazezian and Bull, 2021). Thus, the treatment of symptomatic HO remains an unmet clinical need.

**FIGURE 1:**
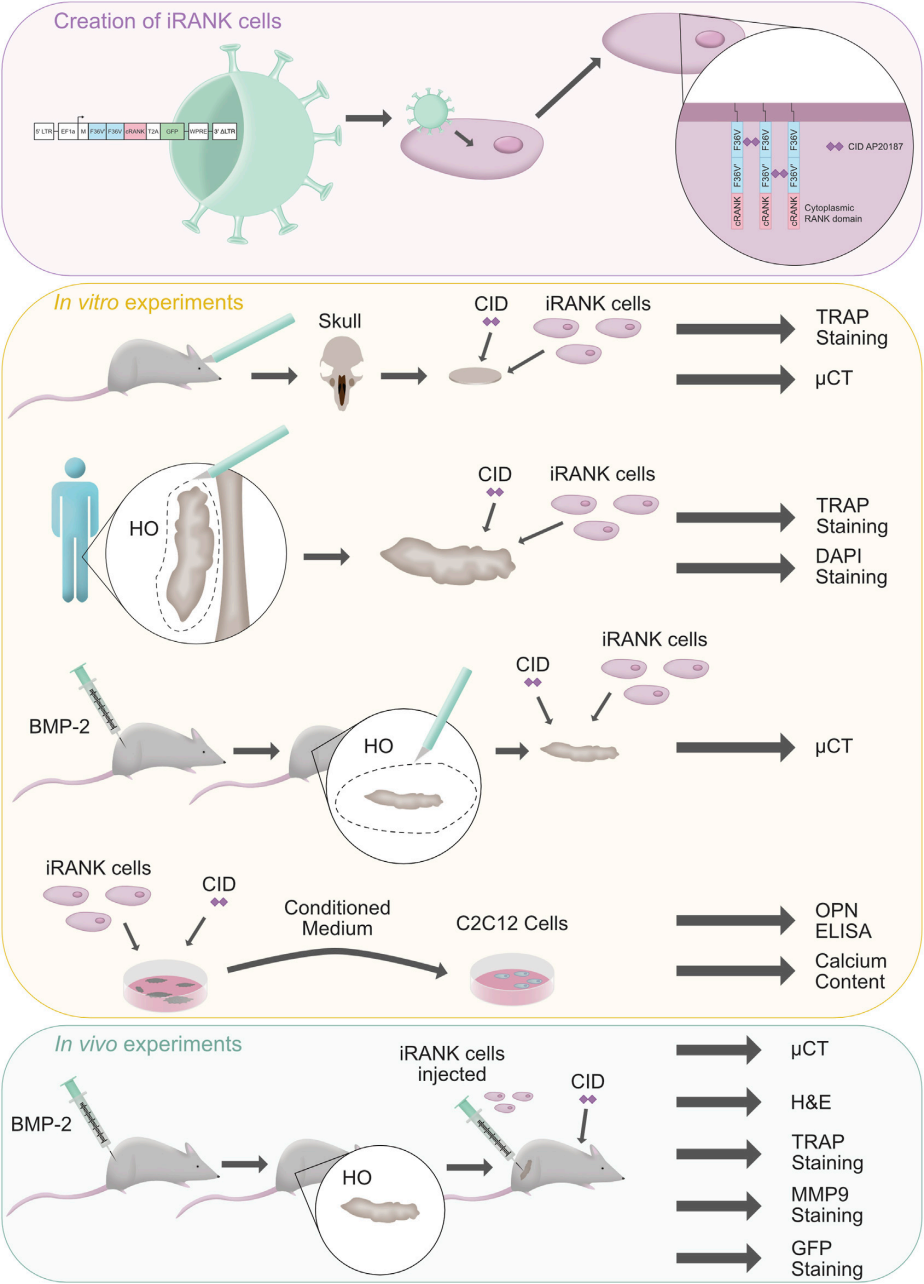
Outline of the study. A schematic summary of the creation of engineered osteoclasts (iRANK cells) is shown with a purple background. These cells were tested within *in vitro* (yellow background) and *in vivo* (green background) experiments. Illustration drawn by Hannah Blümke using Affinity Designer 2.1.1.

Cell therapy has been proposed as a potential treatment for HO since delivery of ectopic bone resorbing cells could, in theory, cause fewer complications than surgical excision (Jin et al., 2021). Osteoclasts (OCs) are physiological bone resorptive cells, but their difficulty in isolation, relatively poor migration and adhesion to ectopic bone, and ability to be inhibited by osteoprotegerin (OPG) limit the use of native OCs for HO cell therapy (Collin-Osdoby and Osdoby, 2012; Rementer et al., 2013; Jin et al., 2021). Native OCs differentiate from monocyte/macrophage precursors through the action of receptor activator of nuclear factor kappa-Β ligand (RANKL) on its cognate receptor, RANK (Rementer et al., 2013; Owen and Reilly, 2018). We previously engineered myeloid precursors that differentiated into OCs in the presence of a small molecule chemical inducer of dimerization (CID) (Rementer et al., 2013). These engineered OCs were multinucleated, tartrate-resistant acid phosphatase (TRAP) and green fluorescent protein (GFP)-positive, capable of robustly resorbing mineralized surfaces *in vitro* (Rementer et al., 2013) and necrotic bone *in vivo* (Buranaphatthana et al., 2021). Crucially, the differentiation of engineered OCs and resorption of bone was RANKL- and OPG-independent (Rementer et al., 2013). The goal of the present study was to further evaluate and test the adherence of engineered OCs to and resorption of HO *in vitro* and *in vivo*. A schematic summary of the study is outlined in Figure 1.

## 2 Materials and methods

### 2.1 Cell culture

RAW264.7 cells were obtained from ATCC (Manassas, VA) and engineered to contain an inducible RANK receptor (iRANK) as previously described (Rementer et al., 2013; Buranaphatthana et al., 2021). In brief, RAW264.7 cells were transduced using a lentiviral vector to express a fusion protein comprising the intracellular domain of RANK and a dimerization domain. The dimerization domain binds to a CID by which dimerization of the construct (including the intracellular RANK domain) can be achieved. OC differentiation and activation are thus initiated via a consecutive downstream signal following dimerization of the intracellular RANK domain. This mechanism is dependent on CID administration and allows for RANKL independent OC differentiation and activation and, in principle, allows for improved control of iRANK cells in *in vivo* environments.

Engineered RAW264.7 cells (iRANK cells) were cultured in α-minimum essential medium (α-MEM) (Invitrogen, Carlsbad, CA) containing 10% (v/v) heat-inactivated fetal bovine serum (FBS) and 1% (v/v) penicillin/streptomycin (P/S) (10,000 U/mL stock solution; Invitrogen, Waltham, MA) and incubated at 37°C with 5% CO_2_. OC differentiation was performed using AP20187 (CID) (Clontech Laboratories, Mountain View, CA) as detailed in the following experiments.

C2C12 mouse myoblast cell line was acquired from ATCC (Manassas, VA) to determine the effects of iRANK CM on the osteoblastic and mineralization capacity of the C2C12 line. C2C12 cells were cultured in Dulbecco’s Modified Eagle Medium (DMEM) (Thermo Fisher Scientific, Waltham, MA) supplemented with 10% (v/v) FBS and 1% (v/v) P/S. Incubation was performed at 37°C in a humidified atmosphere containing 5% CO_2_.

### 2.2 *In vitro* assessment of engineered osteoclasts

#### 2.2.1 Assessment of engineered osteoclasts on calvarial disks

##### 2.2.1.1 Collection and preparation of calvarial disks

Murine calvarial disks were obtained from 4 to 5-week-old wild-type C57BL/6 mice. After mice were euthanized with carbon dioxide, an incision was made over the skull. The roof of the skull was excised with surgical scissors and cleaned of adherent tissue using a sterile cotton swab. Two calvarial disks were obtained from each mouse using a 5 mm biopsy punch. These were first rinsed in a solution of phosphate buffered saline (PBS) (Gibco, Waltham, MA) containing 1% (v/v) antibiotic-antimycotic (10,000 U/mL P/S, 25 μg/mL Amphotericin B stock solution; Gibco, Waltham, MA) twice. The disks were frozen in solution overnight to aid in cell lysis. Each disk was placed in a vial containing 5 mL of 70% (v/v) ethanol and then sonicated (Sonic Disruptor TM-500, Tekmar) at an amplitude of 90 microns for 5 min twice. The disks were then rinsed in sterile water twice and dried in a lyophilizer [disks were dried in a lyophilizer (Virtis Benchtop Lyophilizer, Viritis, Warminser, PA) overnight]. Collected and prepared calvarial disks were then assessed by micro-CT (µCT) as described below.

##### 2.2.1.2 Cell seeding and osteoclast differentiation on calvarial disks

Following µCT image acquisition, disks were cleaned again by rinsing in PBS containing 1% antibiotic-antimycotic, followed by 70% (v/v) ethanol and finally sterile water. Disks were incubated in α-MEM containing 10% (v/v) FBS for 1 h prior to cell seeding. After 1 h, media was removed, and the disks were allowed to air dry for 10 min iRANK cells were seeded at 100,000 cells per disk (5 mm diameter) on one side in 100 µL of α-MEM. The disks were placed in the incubator for 1 h to allow cell adhesion before being seeded on the other side of the disk in a similar manner. For this purpose, disks were placed into new wells with the just seeded side facing down. Then, iRANK cells were seeded with the same concentration of cells on the side that was facing up. The cells were allowed to adhere by placing the disk in the incubator again for 1 h before replacing the α-MEM with or without the addition of 50 nM of the CID AP20187. Disks remained in culture with media changes every 2–3 days. Disks were analyzed on day 12. Following µCT image acquisition, the disks were cleaned, reseeded and treated with iRANK cells for 10 more days as described above. Final scans were taken on day 22.

##### 2.2.1.3 Assessment of osteoclast differentiation using TRAP staining

In order to confirm OC adhesion and differentiation on calvarial disks, TRAP staining was performed after 22 days of differentiation. For this, calvarial disks were washed twice with PBS, fixed with 10% (v/v) neutral buffered formalin (Electron Microscopy Sciences, Hatfield, PA) and incubated in TRAP staining solution for 30 min at 37°C according to the manufacturer’s instructions (387A, Sigma-Aldrich, St. Louis, MO). Images were acquired using an upright microscope in brightfield mode (Nikon E800, Tokyo, Japan).

##### 2.2.1.4 Identification of engineered osteoclasts using fluorescence microscopy

As mentioned above, a GFP reporter gene was included in the iRANK construct in order to differentiate and identify iRANK cells form other cells. In addition to sampling calvarial disks for TRAP, disks were analyzed for GFP signals using an upright fluorescence microscope (Nikon E800, Tokyo, Japan).

##### 2.2.1.5 Assessment of HO regression using microcomputed tomography

µCT was used to assess the bone volume before and after treatment with iRANK cells. The disks were scanned using a µCT (Scanco VivaCt 40; 10.5-μm voxel size, 55 kVp, 145 μA) as previously described (Ausk et al., 2015) and the bone volume was calculated using a threshold of 250 mg hydroxyapatite (HA)/cm^3^. Throughout the experiments, two calvarial disks not used for treatment with iRANK cells were set aside to be scanned with every batch of disks used for iRANK cell treatment. This was done to ensure that measurements were consistent throughout every batch, increasing the reliability of the measurements.

After the scans were obtained, an automated contouring algorithm was used to isolate HO volumes as previously described (Ausk et al., 2015). Both total tissue volume (defined by the perimeter of HO lesion) and bone volume (calcified volume within the total tissue volume) were measured. A threshold of 250 mg HA/cm^3^ was chosen to identify calcified tissue.

#### 2.2.2 Assessment of engineered osteoclasts on human HOs

##### 2.2.2.1 Collection and preparation of human HOs

Samples of discarded, deidentified human HOs were obtained following HO resection surgery (Dept. of Orthopedic Surgery, University of Washington, Seattle, WA). Human HO samples were stored in 70% (v/v) ethanol before being sectioned into 300 μm slices using a precision saw (Buehler Isomet, Buehler, Lake Bluff, IL) equipped with a diamond wafering blade. The sectioned HOs were sonicated in 70% (v/v) ethanol, ddH_2_O, and α-MEM media supplemented with 10% (v/v) FBS and 1% (v/v) P/S for 10 min in each condition, respectively. HOs were incubated in α-MEM media with 10% (v/v) FBS and 1% (v/v) P/S at 37°C for ∼2 h before cell seeding.

##### 2.2.2.2 Cell seeding and osteoclast differentiation on human HOs

To study the adhesion of iRANK cells to the HOs, the sesctioned HOs were placed at the bottom of the wells of a 24-well plate. A total of 2 × 10^4^ iRANK cells were seeded per well on top of the HO in DMEM media with 10% (v/v) FBS and 1% (v/v) P/S. After 6 h, the unattached cells and media were removed. The attached iRANK cells were then treated with 10 nM CID in α-MEM media supplemented with 10% (v/v) FBS and 1% (v/v) P/S for 5 days to induce OC differentiation. The media and treatment were changed once on day 3.

##### 2.2.2.3 Assessment of engineered osteoclasts on human HOs by TRAP and nuclei staining

On day 5, the adhered iRANK OCs and HOs were washed twice with PBS, fixed with 10% (v/v) neutral buffered formalin for 10 min, and incubated with TRAP staining solution for 30 min at 37°C according to the manufacturer’s protocol as described above. Nuclei were counterstained with 300 nM DAPI (Life Technologies, Carlsbad, CA) for 5 min. Following staining, the HO nodule was placed in an imaging chamber with a glass coverslip bottom (80427, Ibidi, Martinsried, Germany) in ddH_2_O. Images were acquired on a scanning confocal microscope (Leica SP8X, Wetzlar, Germany) with a water immersion objective. Images were captured with a transphoton multiplier tube (PMT) detector for TRAP staining and a hybrid (HyD) detector for wavelengths of 421–508 nm for DAPI staining.

#### 2.2.3 Assessment of engineered osteoclasts on mouse HO nodules

##### 2.2.3.1 Formation, collection and preparation of mouse HO nodules

In order to induce the formation of HOs in C57BL/6 mice, mice were injected with 2.5 µg of BMP-2 dissolved in 20 µL of Cultrex Basement Membrane Extract (BME) (R&D Systems, Minneapolis, MN) into the belly of the right calf muscle under ketamine/xylazine anesthesia. After 4 weeks, the mice were scanned using µ-CT under isoflurane anesthesia to locate the induced HO nodule. After scanning, the mice were euthanized using carbon dioxide and the HO nodules were dissected from the surrounding tissue and frozen overnight, before being prepared for cell seeding in the same manner as the calvarial disks described above.

##### 2.2.3.2 Cell seeding and osteoclast differentiation on mouse HO nodules

Excised and prepared murine bone nodules were placed in 96-well plates and seeded with 100,000 iRANK cells in α-MEM supplemented with 10% (v/v) FBS and 1% P/S. After 1 h, the samples were inverted, and the next surface was seeded with cells. This procedure was repeated until the surfaces of the HO samples were coated with cells. After 1 h, the media was replaced with media with or without 50 nM of the CID AP20187. The nodules were reseeded with fresh cells after 12 days and cultured for a total of 22 days after the initial cell seeding. Quantitation of the calvarial disk bone volume was performed by µ-CT as described above.

#### 2.2.4 Assessment of inhibition of passive mineralization by engineered osteoclasts

##### 2.2.4.1 Creation of osteoclast conditioned media

The below stated findings indicate that a regression in HO might not only be due to iRANK OC resorption, but also due to inhibition of passive mineralization. In order to investigate whether iRANK OCs secrete osteopontin (OPN), a potent agent that inhibits passive mineralization (Wang et al., 2021), conditioned media (CM) was created. For this purpose, iRANK cells were cultured in α-MEM containing 10% (v/v) heat-inactivated FBS and 1% (v/v) P/S in the absence or presence of 50 nM of the CID AP20187 to induce OC differentiation. On day 5, the media was replaced with DMEM containing 3% (v/v) heat-inactivated FBS and 1% (v/v) P/S. CM was collected, centrifuged to remove cells and filtered through a 0.22 µM filter (Fisher Scientific, Hampton, NH). CM was collected daily for 4 days and stored at −20°C.

##### 2.2.4.2 Osteopontin enzyme-linked immunosorbent assay

CM was subjected to OPN enzyme-linked immunosorbent assay (ELISA) following the manufacturer’s instructions (Mouse Osteopontin DuoSet, R&D Systems, Minneapolis, MN). Briefly, polystyrene flat-bottomed microtiter plates (Thermo Fisher Scientific, Waltham, MA) were coated with 0.8 μg/mL of capture antibody (goat anti-mouse OPN antibody) (Mouse Osteopontin DuoSet, R&D Systems, Minneapolis, MN) in PBS overnight at room temperature. The wells were washed three times with wash buffer, consisting of 0.05% (v/v) Tween 20 in PBS, and were blocked with reagent diluent, consisting of 1% (w/v) bovine serum albumin (BSA) in PBS. After washing the plates three times, twofold serial dilutions of standard in reagent diluent (15.625-1,000 pg/mL of OPN) and CM samples (diluted in reagent diluent) were added in duplicates to the wells. Media was used as a negative control. The microplates were incubated for 2 h at room temperature. After washing the plates three times, the detection antibody (biotinylated goat anti-mouse OPN) (Mouse Osteopontin DuoSet, R&D Systems, Minneapolis, MN) was added to each well and incubated for 2 h at room temperature. Following the washing step, streptavidin conjugated to horseradish-peroxidase was added, and incubated for 20 min at room temperature. The wells were washed with wash buffer three times. Substrate solution was added to each well and incubated for 20 min in the dark. Stop solution was added to each well and finally the optical density of each well was measured using a microplate reader set at 450 nm excitation and 540 nm emission.

##### 2.2.4.3 Immunodepletion of OPN from conditioned media

Dynabeads Protein G (1.5 mg) (Life Technologies, Carlsbad, CA) were incubated with OP-199 antibody (Liaw et al., 1994; Rittling and Feng, 1998) (10 µg) with rotation for 30 min at room temperature in an Eppendorf tube. The tube was then placed on the magnet and the beads-antibody complex was washed with PBS with Tween 20 twice. CM from CID-treated engineered iRANK cells (1 mL) was added to the Dynabead-antibody complex and incubated with rotation for 1 h at room temperature according to manufacturer’s instruction. The tube was placed on the magnet and CM was then transferred to a clean tube and filtered using a cell strainer.

##### 2.2.4.4 Incubation of C2C12 cells with osteoclast conditioned media

CM was added to C2C12 culture in a 1:1 ratio of CM to fresh media. Next, Na_2_PO_2_/NaPO_2_ (pH equilibrated to 7.4) was added to C2C12 cultures to obtain a final concentration of 3.2 mM inorganic phosphate (Pi). The media was replaced and transferred every day. At day 5 of C2C12 culture, calcium content of C2C12 cells was quantified using the O-cresolphthalein complexone method as previously described (Steitz et al., 2002). Briefly, the cultures were decalcified with 0.6 N HCl overnight. Calcium content in HCl supernatant was determined colorimetrically by the O-cresolphthalein complexone method (C503-480, Teco Diagnostics, Anaheim, CA). After removing the HCl supernatant, the cultures were washed with PBS and solubilized with 0.2 N NaOH. The total protein content was measured with the Micro BCA protein assay kit according to manufacturer’s instructions (Thermo Fisher Scientific, Waltham, MA).

### 2.3 *In vivo* assessment of engineered osteoclasts

#### 2.3.1 *In vivo* mouse HO model

HO formation was induced in nude mice using BMP-2. Mice were anesthetized, and the mid-belly of the gastrocnemius muscle was injected with a mixture of recombinant human BMP-2 (Syd Labs Inc., Boston, MA, United States) and BME at a final concentration of 2.5 µg BMP-2/20 µL of BME. The HO formations were allowed to form for 28 days before treatment with iRANK cells *in vivo*. At day 28, HO formations were scanned by µ-CT in identical manner as mentioned above.

#### 2.3.2 Cell delivery of engineered osteoclasts to HO formations *in vivo*

A total of 5 × 10^6^ iRANK cells in 25 µL of a collagen carrier were injected onto HO formations on day 28 following CT image acquisition. Six mice received iRANK cells within the collagen carrier. Five animals were injected with the collagen carrier without cells. In the cell delivery group, the CID AP20187 was administered intraperitoneally daily for 3 days and then every other day. For injection, CID was formulated with 10% (v/v) PEG 400% and 1.7% (v/v) Tween 20. Animals received 2 mg/kg body weight of the CID AP20187 at each injection (Larrivée et al., 2005). Animals were scanned again after 1 week and the iRANK group received another cell injection. After 2 weeks, a final µ-CT scan was performed, and the entire calf was explanted and fixed in 10% (v/v) buffered formalin for histological examination.

#### 2.3.3 Analysis of HO total volume and bone volume using µCT

µCT was used to assess *in vivo* HO formations before, during, and after treatment with iRANK cells or simply the collagen carrier as a control. For this, mice were anesthetized with isoflurane and scanned using a µCT (Scanco vivaCT 40; 21-μm voxel size, 55 kVP, 145 μA). Total tissue volume and bone volume were analyzed as stated above.

#### 2.3.4 Histological examination using hematoxylin and eosin staining

Following explanation, tissues were first decalcified using a commercial decalcifying solution (Cal-Ex Decalcifier, Fisher Scientific, Hampton, NH) for 1 week before tissue processing and sectioning. For Hematoxylin and Eosin staining (H&E), tissue sections were deparaffinized, rehydrated, and placed in Harris Hematoxylin solution (Sigma-Aldrich, Darmstadt, Merck) for 3 min before rinsing. This was followed by placing samples for 40 s in 0.25% (v/v) ammonium water. Finally, slides were dipped in Eosin solution (Sigma-Aldrich, Darmstadt, Merck) 10 times before dehydrating. The slides were mounted using a mounting medium (Permount, Thermo Fisher Scientific, Waltham, MA). Images were obtained using an upright microscope in brightfield mode (Nikon E800).

#### 2.3.5 Identification of osteoclasts on *in vivo* HO formations using TRAP and DAPI staining

In order to confirm TRAP expression and multinucleation of OCs, tissue sections were deparaffinized, rehydrated and incubated for 30 min with TRAP staining solution at 37°C in an identical manner as mentioned above. Nuclei were counterstained with 300 nM DAPI (Life Technologies, Waltham, MA) for 5 min. Slides were mounted with a mounting medium (Aqua-Mount, Thermo Fisher Scientific, Waltham, MA) and images were obtained using an upright microscope, using either the brightfield mode for image acquisition of TRAP-stained samples or the DAPI mode for DAPI stained samples (Nikon E800, Tokyo, Japan).

#### 2.3.6 Identification of engineered osteoclasts on *in vivo* HO formations and assessment of osteoclast functionality by staining for MMP9, GFP and nuclei

A polyclonal antibody for GFP was used for immunofluorescence staining (A-11122, Thermo Fisher Scientific). Slides were deparaffinized and rehydrated with Tris-buffered saline with Tween (TBST), consisting of 10 mM Tris buffer, 150 mM NaCl, and 0.5% (v/v) Tween-20, equilibrated to a pH of 7.6 prior to antigen retrieval. Slides were incubated in boiling citrate buffer, consisting of 10 mM citrate, and 0.05% Tween 20, equilibrated to a pH of 6 for 10 min and allowed to cool. Blocking was performed with 4% (v/v) normal donkey serum (NDS) with 1% (w/v) BSA in PBS for 1 h before adding the primary antibody. The anti-GFP antibody was diluted in 2% (v/v) NDS and 1% (w/v) BSA at a ratio of 1:400 and incubated at 4°C overnight. Slides were rinsed 3 times in TBST for 5 min each before the secondary antibody was added. Donkey anti-rabbit IgG (Cy3 AffiniPure, Jackson ImmunoResearch Laboratories, West Grove, PA) was diluted in 2% (v/v) NDS and 1% (w/v) BSA at a dilution of 1:500 and incubated for 30 min. Slides were rinsed 3 times in TBST for 5 min each before mounting with a mounting medium (ProLong Gold Antifade Mountant, Invitrogen, Carlsbad, CA). Cells were counterstained with DAPI as described above.

In addition to GFP staining, matrix metalloproteinase-9 (MMP9)/GFP co-staining was utilized to assess iRANK OC activity. For this, slides were deparaffinized and rehydrated with PBS containing 0.1% (w/v) Tween 20 (PBS-T) (Sigma-Aldrich, Darmstadt, Germany), before permeabilization with 0.1% (v/v) Triton X-100 in PBS for 10 min. Blocking was performed with 5% (v/v) NDS in PBS for 1 h. Slides were incubated with primary antibodies including rabbit anti-GFP polyclonal antibody (A-11122, Thermo Fisher Scientific) at a 1:200 dilution and goat anti-MMP9 antibody (AF909, Novus, Littleton, CO) at a 1:100 dilution in 2% NDS in PBS at 4°C overnight. Slides were rinsed 3 times in PBS-T for 5 min. This was followed by incubation with secondary antibodies including a donkey anti-rabbit IgG (Cy3 AffiniPure, Jackson ImmunoResearch Laboratories, West Grove, PA) at a 1:800 dilution and a donkey anti-goat IgG (Alexa Fluor 488 AffiniPure, Jackson ImmunoResearch Laboratories, West Grove, PA) at a 1:500 dilution in PBS at room temperature for 30 min. Slides were then rinsed three times in PBS-T for 5 min and once in ddH_2_O. Nuclei were counterstained with 300 nM DAPI (Life Technologies, Carlsbad, CA) for 5 min. Slides were rinsed with ddH_2_O and PBS before mounting with mounting medium (ProLong Gold Antifade Mountant, Invitrogen, Carlsbad, CA).

Images were obtained using an upright microscope (Nikon E800, Tokyo, Japan) and overlaid using Adobe Photoshop to localize MMP9^+^/GFP^−^ and MMP9^+^/GFP^+^ cells with three or more nuclei indicating endogenous and iRANK OCs, respectively. ImageJ was used for MMP9 quantification (Rueden et al., 2017). A threshold was set to differentiate between specific MMP9 staining and background staining. This threshold was applied to all images. The areas with cells whose intensity surpassed the threshold were labeled as MMP9^+^. The total area of the HO and the total area minus the marrow area within the HO were quantified.

### 2.4 Statistical analysis

Statistical analysis was carried out using GraphPad Prism 9. Statistical significance was assessed using Student’s t-test and ANOVA in conjunction with either Tukey’s or Šídák’s *post hoc* test as indicated in the individual experiments. A *p*-value of *p* < 0.05 (*p < 0.05, **p < 0.01, ***p < 0.001, ****p < 0.0001) was considered to be statistically significant. Biological replicates (n) are provided in the corresponding figure legends.

## 3 Results

### 3.1 Engineered osteoclasts attach to and reduce bone volume on murine calvarial disks as well as murine and human HO nodules *in vitro*

**FIGURE 2:**
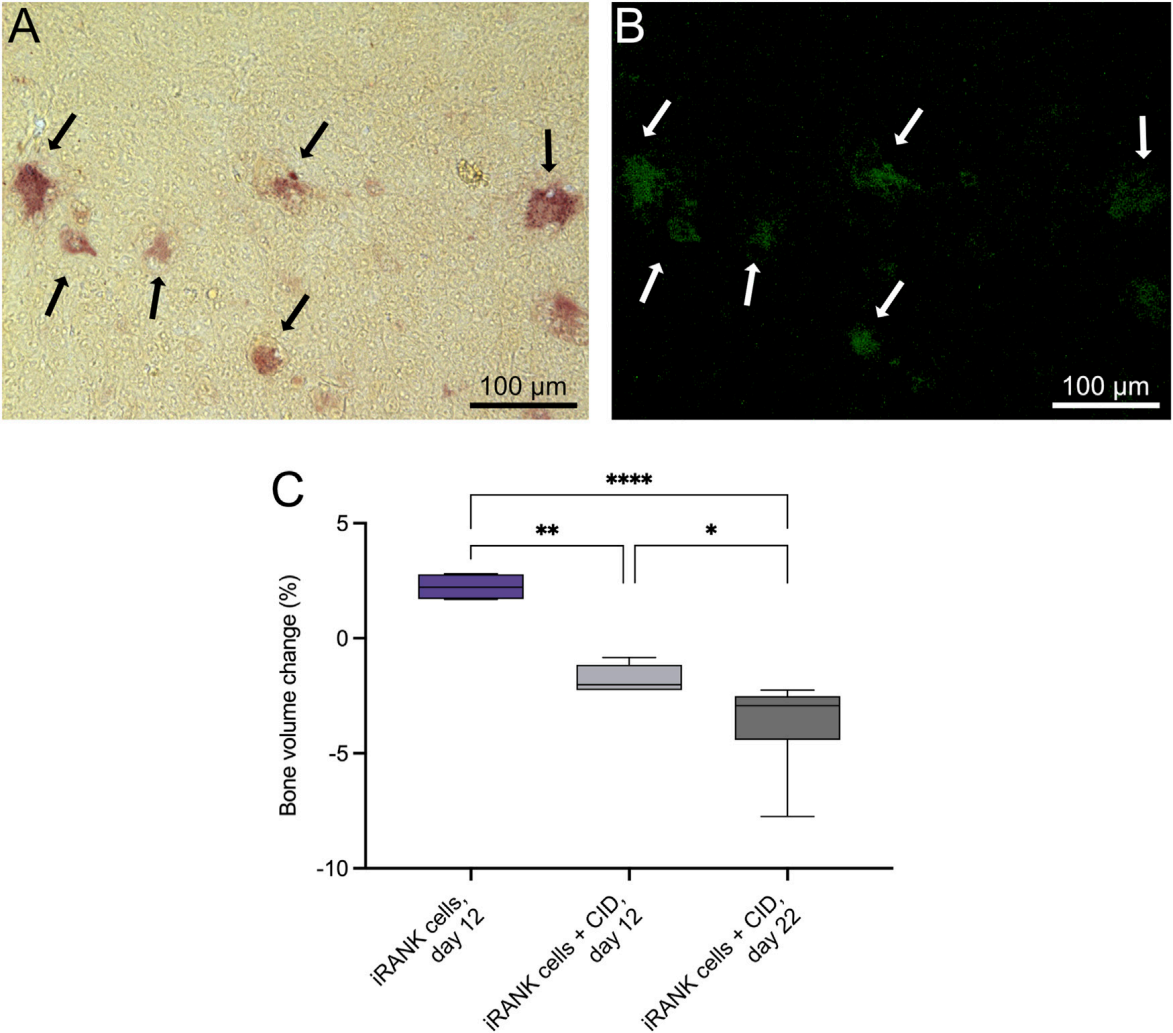
iRANK cells induced with a chemical inducer of dimerization resorb calvarial bone *in vitro*. Following osteoclast differentiation of iRANK cells using a chemical inducer of dimerization, osteoclasts **(A)** stain for TRAP (black arrows), and **(B)** also appear GFP^+^ (white arrows) since engineered iRANK cells contain a GFP domain within the iRANK construct. **(C)** µCT analysis of *in vitro* treated murine calvarial disks showed a significant decrease in the percentage of bone volume change compared to disks treated with iRANK cells that had not been induced using the chemical inducer of dimerization. This decrease could be enhanced by reseeding and continued induction of cells using the chemical inducer of dimerization, as assessed by Šídák’s multiple comparison in conjunction with ANOVA (n = 4–7 donors, *p < 0.05, **p < 0.01, ****p < 0.0001). Scale bar: A, B = 100 µm.

RAW264.7 myeloid precursor cells containing a CID-inducible, intracellular RANK signaling domain (iRANK cells) were developed as previously described (Rementer et al., 2013). We previously showed that iRANK cells differentiate into OCs in the presence of CID and robustly resorb two-dimensional mineralized surfaces *in vitro* (Rementer et al., 2013; Buranaphatthana et al., 2021). To determine whether engineered OCs could resorb healthy three-dimensional bone, murine calvarial bone discs were utilized. The discs were seeded with iRANK cells on both sides and treated with or without CID. TRAP^+^ (Figure 2A) and GFP^+^ (Figure 2B) multinucleated OCs were observed on discs coated with iRANK cells with CID treatment (black and white arrows), while no OCs formed in discs coated with iRANK cells in the absence of CID. We further investigated the bone resorptive ability of these engineered OCs. Discs seeded with iRANK cells and treated with CID showed a significant decrease in bone volume of 1.8% at 12 days and 3.8% at day 22 (Figure 2C). In contrast, discs coated with iRANK cells in the absence of CID showed a small increase in bone volume (Figure 2C), similar to that observed for passive mineralization in cell-free media containing calcium and phosphate (Nguyen et al., 2023).

**FIGURE 3:**
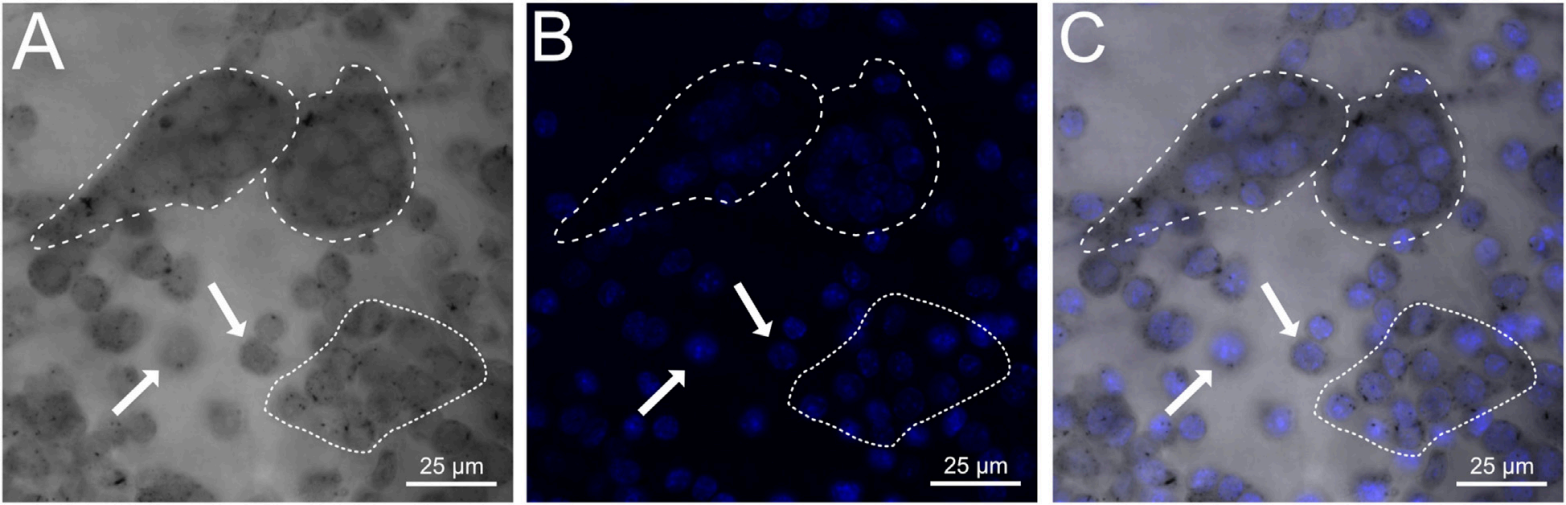
iRANK cells adhere to and differentiate into osteoclasts on human heterotopic ossifications. **(A)** Brightfield confocal imaging showing diffuse as well as punctate cytoplasmic TRAP staining in mononucleated pre-osteoclasts (white arrows) and multinucleated osteoclasts (dashed white line). Preosteoclasts in the process of fusion are outlined by a dotted white line. **(B)** DAPI staining confirms multinucleation of osteoclasts (dashed white line), which are also depicted additionally in a **(C)** merged image. Scale bar = 25 µm.

To determine whether engineered OCs could attach to and differentiate on ectopic bone samples, iRANK cells were seeded onto human HO surgical specimens and treated with CID. iRANK cells treated with CID successfully adhered to, differentiated and fused into TRAP^+^, multinucleated OCs as seen by TRAP staining and DAPI staining in Figures 3A, B, respectively (outlined by dashed white line). Some single mononucleated, TRAP^+^ (white arrows) as well as clusters of TRAP^+^ mononuclear iRANK cells (dotted white line), likely in the process of fusing into multinucleated OCs, were also observed adherent to the human HO samples.

**FIGURE 4:**
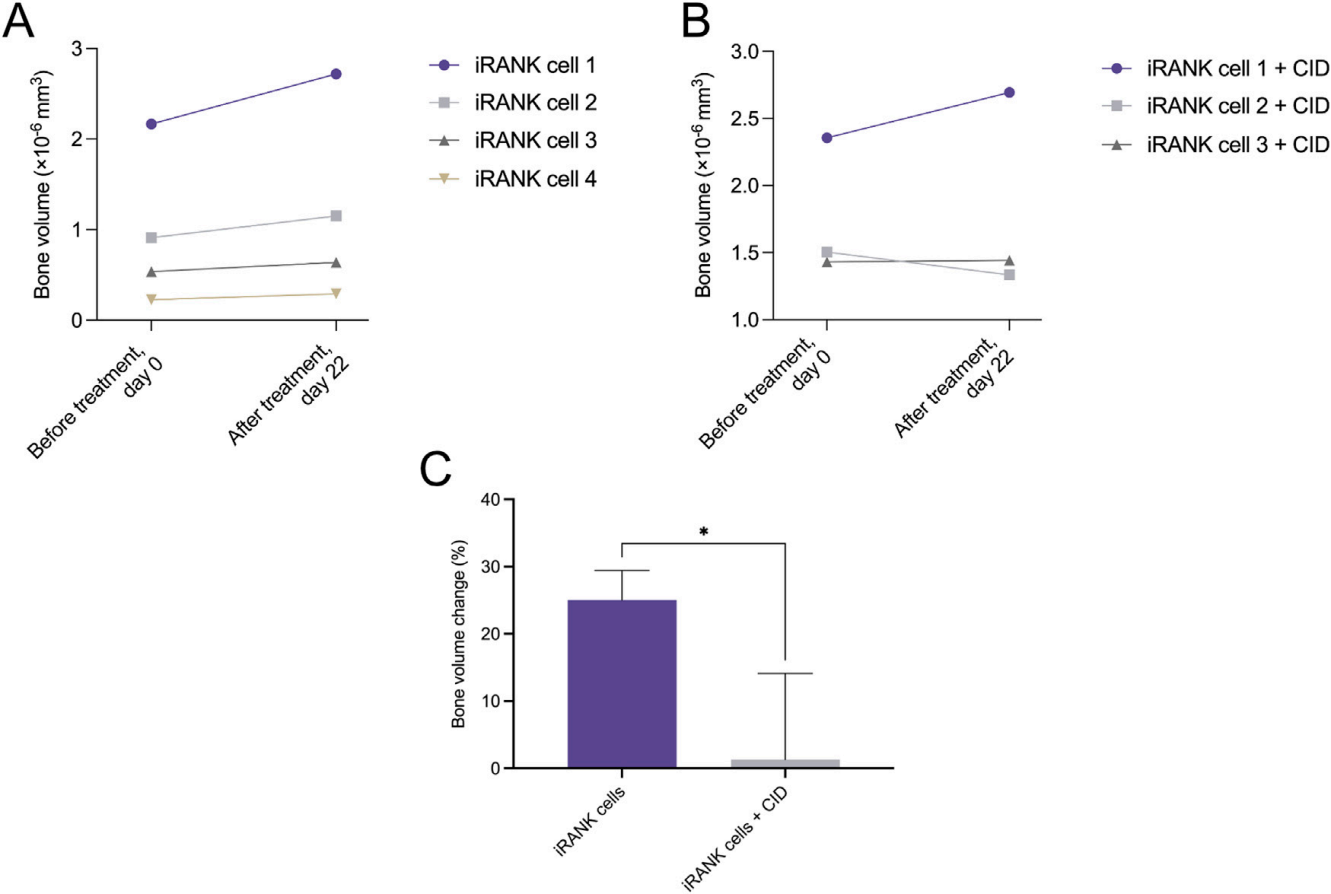
Murine heterotopic ossification nodules significantly decreased in the percentage of bone volume change in response to treatment with activated iRANK cells. **(A)** Overall, an increasing trend in bone volume can be observed for nodules seeded with iRANK cells, but not activated using the chemical inducer of dimerization, probably due to passive mineralization. **(B)** When differentiating and activating iRANK cells using the chemical inducer of dimerization two out of three cell groups show a decreasing trend in relation to bone volume. **(C)** Data expressed as the percentage of bone volume change demonstrate a statistically significant reduction, as assed by Student’s t-test (n = 3–4, *p < 0.05).

To determine whether engineered OCs could resorb ectopic bone, HO nodules were explanted from mice 3 weeks following BMP-2 injection into the calf. HO nodules were treated with iRANK cells and CID or iRANK cells without CID *in vitro* starting at day 0. Treatment was repeated at day 12. Similar to calvarial bone (Figure 2) and human HO (Figure 3), engineered OCs adhered to and differentiated on mouse HO nodules in the presence of CID. As shown in Figure 4A, none of the mouse HO nodules treated with cells and no CID showed any evidence of mineral regression after treatment. In contrast, one of the three HO nodules treated with iRANK cells and CID showed some evidence of regression while the bone volume of the third group stayed constant (Figure 4B). A significant overall decline in the percentage of bone volume change was observed in this group compared to cells not treated with CID (Figure 4C). However, passive mineralization was much higher in mouse HO samples compared to calvarial bone (25.03% as seen in Figure 4C, vs. 2.24%, as seen in Figure 2C), making true resorption more difficult to distinguish from inhibition of passive mineralization. Thus, a decrease in bone volume observed in HOs treated with iRANK cells and CID might be due to a combination of mineral resorption and inhibition of passive mineralization.

### 3.2 Engineered osteoclasts secrete functional osteopontin

OCs have previously been reported to inhibit bone formation (Daponte et al., 2024), suggesting they might secrete potent mineralization inhibitors. OPN is a secreted, phosphorylated glycoprotein that regulates bone mineralization by tightly binding to HA, inhibiting HA crystal growth, and providing adhesion sites for OC resorption of bone (Scatena et al., 2007). In addition, OPN promotes OC function through the ligation of the α_v_β_3_ integrin (Giachelli et al., 2005; Scatena et al., 2007). Previous studies showed the upregulation of OPN expression in two-dimensional gel electrophoresis during OC differentiation (Kubota et al., 2003). This suggests that OPN secretion is elevated during OC differentiation to block HA crystal growth and provide myeloid precursor and OC adhesion sites on mineralized tissues.

**FIGURE 5:**
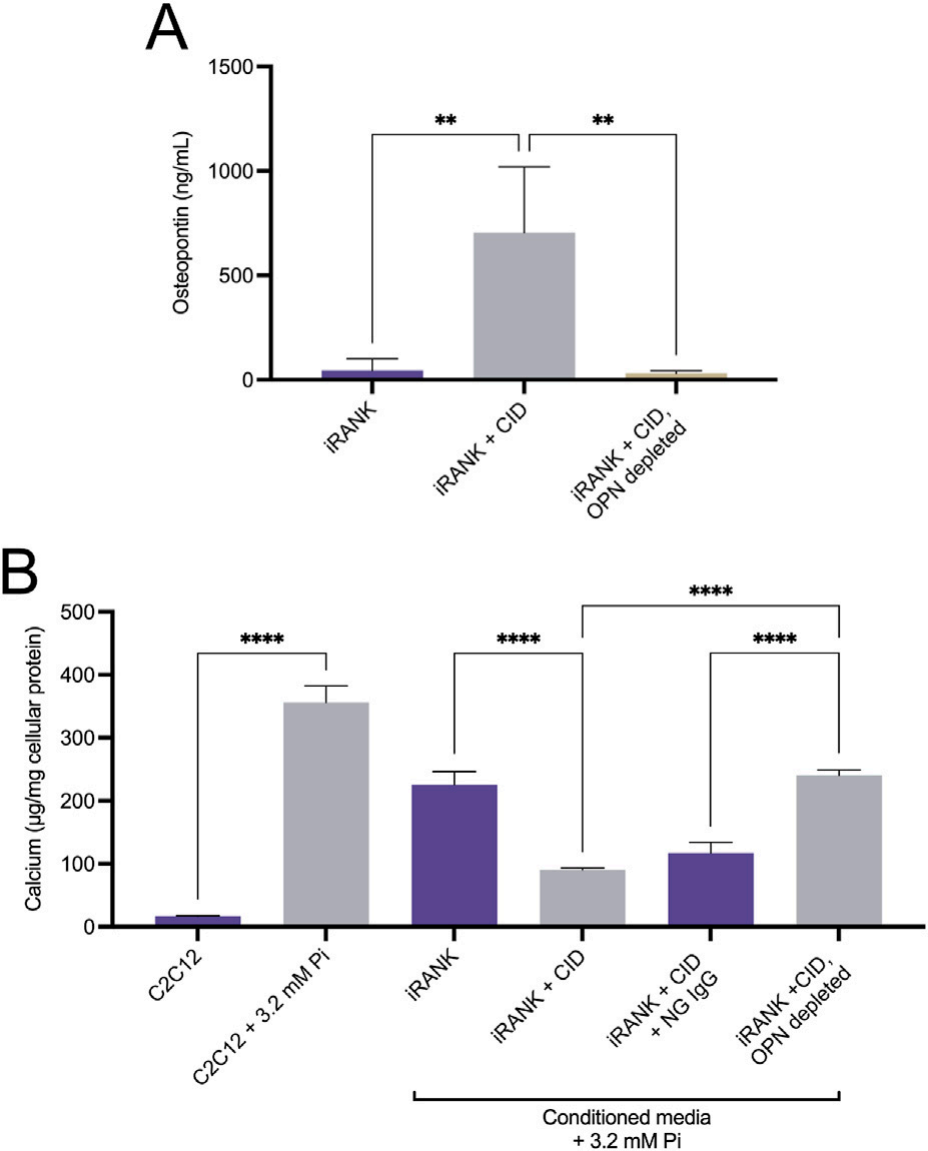
Functional, phosphorylated OPN is highly secreted by engineered osteoclasts. **(A)** iRANK cells were cultured in the absence or presence of 50 nM chemical inducer of dimerization to induce osteoclast differentiation. ELISA was used to detect osteopontin (OPN) levels in the culture media. Untreated iRANK cells secreted significantly less OPN than iRANK cells induced with the chemical inducer of dimerization. OPN was successfully depleted from conditioned media using an OP-199 antibody, as assessed by Tukey’s multiple comparison in conjunction with ANOVA (n = 3–4, **p < 0.01). **(B)** Conditioned media was transferred every day to C2C12 cells in the presence of 3.2 mM inorganic phosphate for 5 days. Conditioned media from iRANK cells induced with the chemical inducer of dimerization inhibited C2C12 calcification. Removal of OPN with OP-199 antibody eliminated the inhibitory effect of the conditioned media from iRANK cells induced with the chemical inducer of dimerization. Normal goat IgG (NG) was used as an isotype control for OP-199 antibody, as assessed by Tukey’s multiple comparison in conjunction with ANOVA (n = 3, ****p < 0.0001).

OPN protein levels in the medium of iRANK cells were significantly higher after 5 days of differentiation into OCs with CID treatment, compared to cells without CID addition (Figure 5A). Furthermore, OPN was efficiently depleted from media using an anti-OPN antibody (Figure 5A). Since OPN absolutely requires phosphorylation to target, block HA crystal growth, and expose arginine-glycine-aspartate-containing adhesion sites (Jono et al., 2000; Scatena et al., 2007), we tested whether the OPN secreted by engineered OCs was functional.

Muscle-derived precursors, C2C12 cells, have been utilized as an *in vitro* model for osteoblastic differentiation and mineralization of mesenchymal precursors in HO (Kikkawa et al., 2009). C2C12 cells grown in media containing 3.2 mM Pi for 5 days underwent osteogenic differentiation and matrix mineralization as previously described (Kikkawa et al., 2009). Quantitation of matrix calcium content demonstrated a statistically significant increase in calcification of C2C12 cells in 3.2 mM Pi compared to cells grown in 1 mM Pi media (Figure 5B). Less mineralization was observed in C2C12 cells treated with 3.2 mM Pi to which iRANK CM was added that did not contain CID during incubation, compared to 3.2 mM Pi alone, likely due to differences in media composition following addition of the CM. Adding iRANK CM that did contain CID during incubation and differentiation resulted in an almost complete blockage of mineral formation. Importantly, depletion of OPN from the CM with a specific antibody that binds mouse OPN removed the inhibitory effect of the CM in C2C12 cultures. These studies confirmed that iRANK OCs secreted functional, phosphorylated OPN.

### 3.3 Engineered osteoclasts attach to and reduce bone volume of HO formations *in vivo*

To investigate whether engineered OCs could resorb HO formations *in vivo*, a cell delivery system and immunotolerant HO mouse model were developed. In order to locally deliver iRANK cells to sites of HO, a delivery vehicle that retained cells at the site of injection and did not interfere with CID-mediated differentiation was required. We confirmed that the collagen gel did not interrupt CID-induced osteoclastogenesis *in vitro* and retained iRANK cells at the HO site *in vivo* (Supplementary Figure S1). Thus, a collagen gel was utilized as a cell carrier in this study.

To establish an *in vivo* HO model, we utilized nude mice to allow for the transplantation of allogeneic iRANK cells. To our knowledge, the ability of HO to form in nude mice following BMP-2 injection has not yet been investigated. Therefore, the ability of HO to form in nude mice within 3 weeks following BMP-2 injection into the calf muscle was examined. Serial µCT showed that mineralized formations were formed at the mid-belly of the calf muscle group over time (Supplementary Figure S2). Mineralizing ectopic bone formations (orange) were located in the mid-belly of the gastrocnemius muscle group, posterior of the tibia and medial to the fibula. At day 28, the average nodule volume was 10,911 μm^3^ and the bone volume normalized by total tissue volume (BV/TV) was 54.2%, similar to findings in wild-type mice (Ausk et al., 2015). No statistically significant changes were observed for both BV/TV ratio and bone mineral density (BMD) between the treatment and control group (Supplementary Figure S3).

**FIGURE 6:**
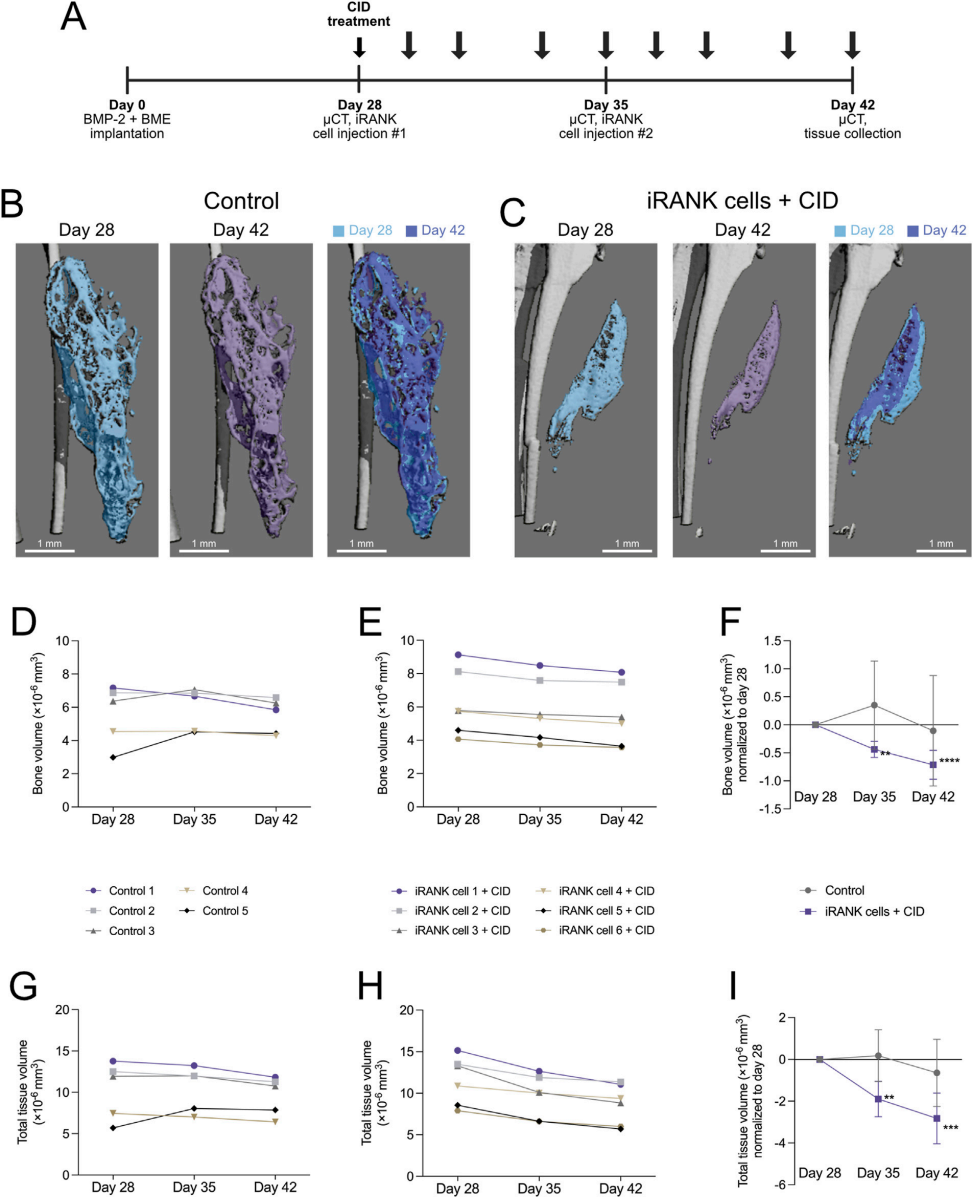
Resorption of HO formations by engineered osteoclasts *in vivo*. **(A)** Experimental timeline showing cell delivery, chemical inducer of dimerization injection, and µCT time schedules following induction of HO formations in calf muscle with BMP-2 and a basement membrane extract (BME). **(B)** µCT images show HO formations, which appear stable with little change in overall morphology over time in the control group, while **(C)** a significant decrease in the size of the HO formation could be seen following treatment using iRANK cells and the chemical inducer of dimerization. **(D)** A stable trend in bone volume can be seen in the control group, while **(E)** animals injected with iRANK cells and the chemical inducer of dimerization depict a decreasing trend. **(F)** When normalized to day 28, a statistically significant decrease can be seen in the treatment group as assessed by Tukey’s multiple comparison in conjunction with ANOVA (n = 5–6, **p < 0.01, ****p < 0.0001). **(G)** The total tissue volume was also stable with only slight decreases in the control group while **(H)** a larger decrease in total tissue volume could be seen in the treatment group. **(I)** When normalized to day 28, a significant decrease could also be observed regarding total tissue volume in the treatment group, as assessed by Tukey’s multiple comparison in conjunction with ANOVA (n = 5–6, **p < 0.01, ***p < 0.001).

Next, we locally delivered iRANK cells to the HO site to determine whether engineered OCs could resorb HO *in vivo*. The overall experimental design is shown in Figure 6A. In brief, iRANK cells were injected at sites of HO formations at day 28 and 35 after inducing formations using BMP-2. CID was injected intraperitoneally throughout the experiment to induce OC differentiation of iRANK cells. µCT was performed at day 28, 35, and 42. Representative µCT images of the control and treatment group are shown in Figures 6B, C, respectively. The differences in the size of HO formations are shown in a purple (day 42) and blue (day 28) overlay for both control and treatment group. No significant visual reduction in the size of the HO formation in the control group can be seen (Figure 6B). In contrast, the HO formation treated with iRANK cells and CID showed a visible reduction in size at day 42 over day 28 (Figure 6C).

Quantitation of µCT scans of the control group (Figure 6D) did not show a clear trend in bone volume over time with relatively stable HO formations, while the treatment group showed an overall trend towards decrease in bone volume as indicated in Figure 6E. When normalized to day 28, a significant decrease of bone volume in the treatment group could be observed (Figure 6F). Similar to previous studies (Ausk et al., 2015), the total volume was also stable, with only slight decreases in the control group (Figure 6G). In contrast, a greater decrease in total volume could be seen in the treatment group (Figure 6H). When normalized to day 28, a significant decrease could also be observed regarding total volume in the treatment group (Figure 6I).

Overall, significant HO regression was detectable after 1 week of iRANK and CID treatment and regression of HO volume continued during the second week of treatment, whereas no significant changes were observed in the control group.

### 3.4 Engineered osteoclasts on HO formations express MMP9 *in vivo*

**FIGURE 7:**
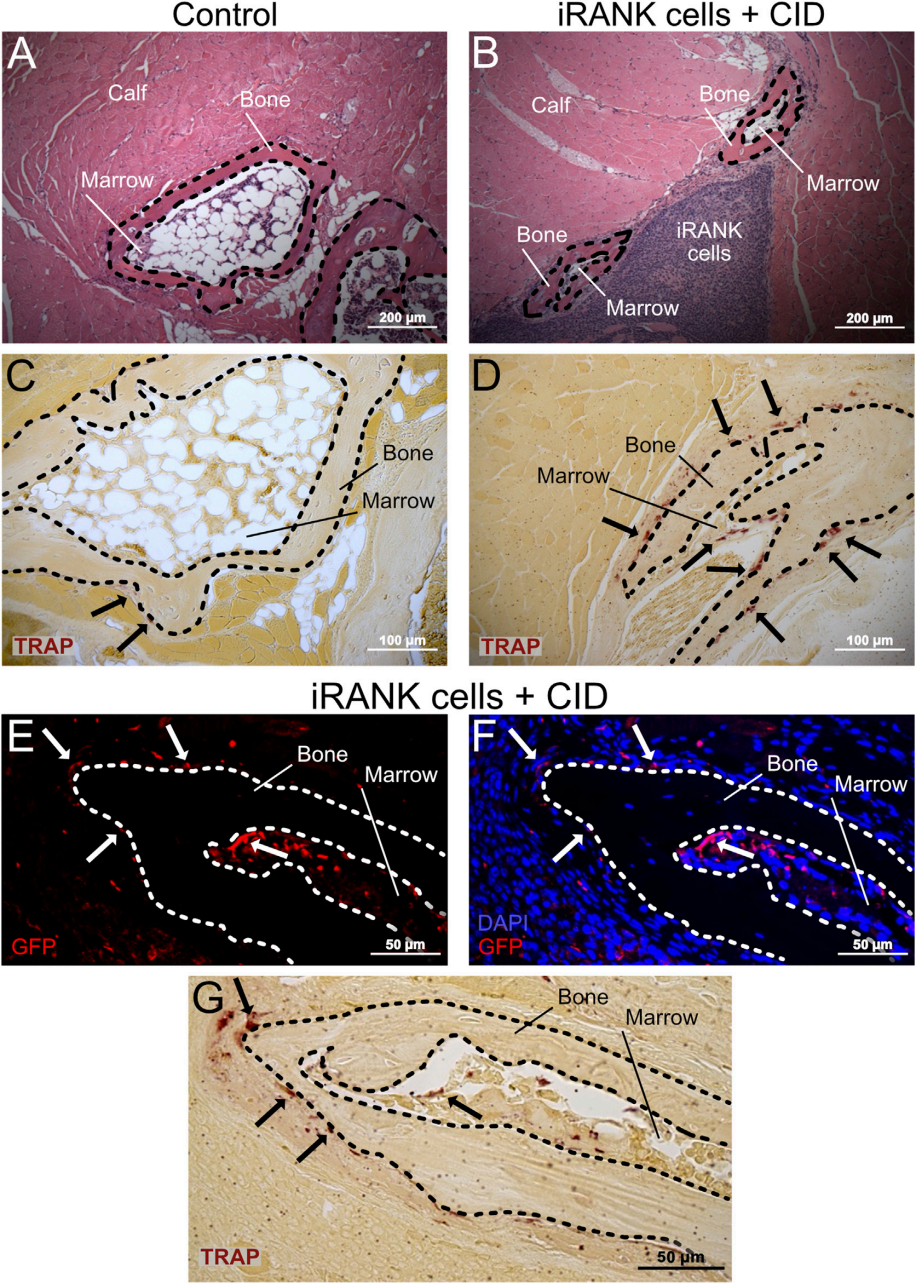
Large numbers of engineered OCs were observed on HO formations in animals treated with iRANK cells and the chemical inducer of dimerization. H&E staining shows HO formations exhibiting a ring-like structures with a mineralized surface (B = Bone) surrounding a marrow-like cavity (M = Marrow) in the **(A)** control group as well as the **(B)** treatment group. **(C)** A greater number of TRAP^+^ osteoclasts (black arrowheads) were observed lining the HO formations in the treatment group compared to the **(D)** control group. **(E)** Immunofluorescence staining for GFP (in red) indicates that many of the **(F)** multinucleated cells (nuclei in blue) lining the HO formations were derived from locally delivered iRANK cells in the iRANK and chemical inducer of dimerization treatment group. **(G)** These GFP^+^ multinucleated cells were also TRAP^+^ as shown in an adjacent tissue section. Dashed lines outline bone tissue. Scale bar: **(A, B)** = 200 μm, **(C, D)** = 100 μm, **(E–G)** = 50 µm.

Following analysis of HO formations using µCT, mice were sacrificed on day 42 for histological analysis. H&E and TRAP staining were performed to determine the morphology of HO formations and to locate OCs within and around the HO formations. HO formations were clearly observed as nodular, bony structures surrounding a marrow-like cavity in both control and iRANK cell treated with CID groups (Figures 7A, B). TRAP^+^ cells were scarce in the formations of the control group, whereas multinucleated TRAP^+^ cells were easily observed in the HO formations treated with iRANK cells and CID (black arrows). OCs in both groups were predominantly located near bone surfaces, consistent with OCs resorbing the lesions (Figures 7C, D).

To confirm that OCs observed on HO formations treated with iRANK cells and CID are derived from delivered engineered iRANK myeloid precursors, we performed immunofluorescence staining for GFP and nuclei in addition to TRAP staining. As expected, GFP^+^ cells were observed at the delivery sites, surrounding the HO as well as in marrow-like spaces (Figures 7E, F). Several multinucleated GFP^+^ cells were observed found lining the bone surfaces (white arrows). Consecutive TRAP staining revealed that these cells were TRAP^+^ (black arrows) (Figure 7G).

**FIGURE 8:**
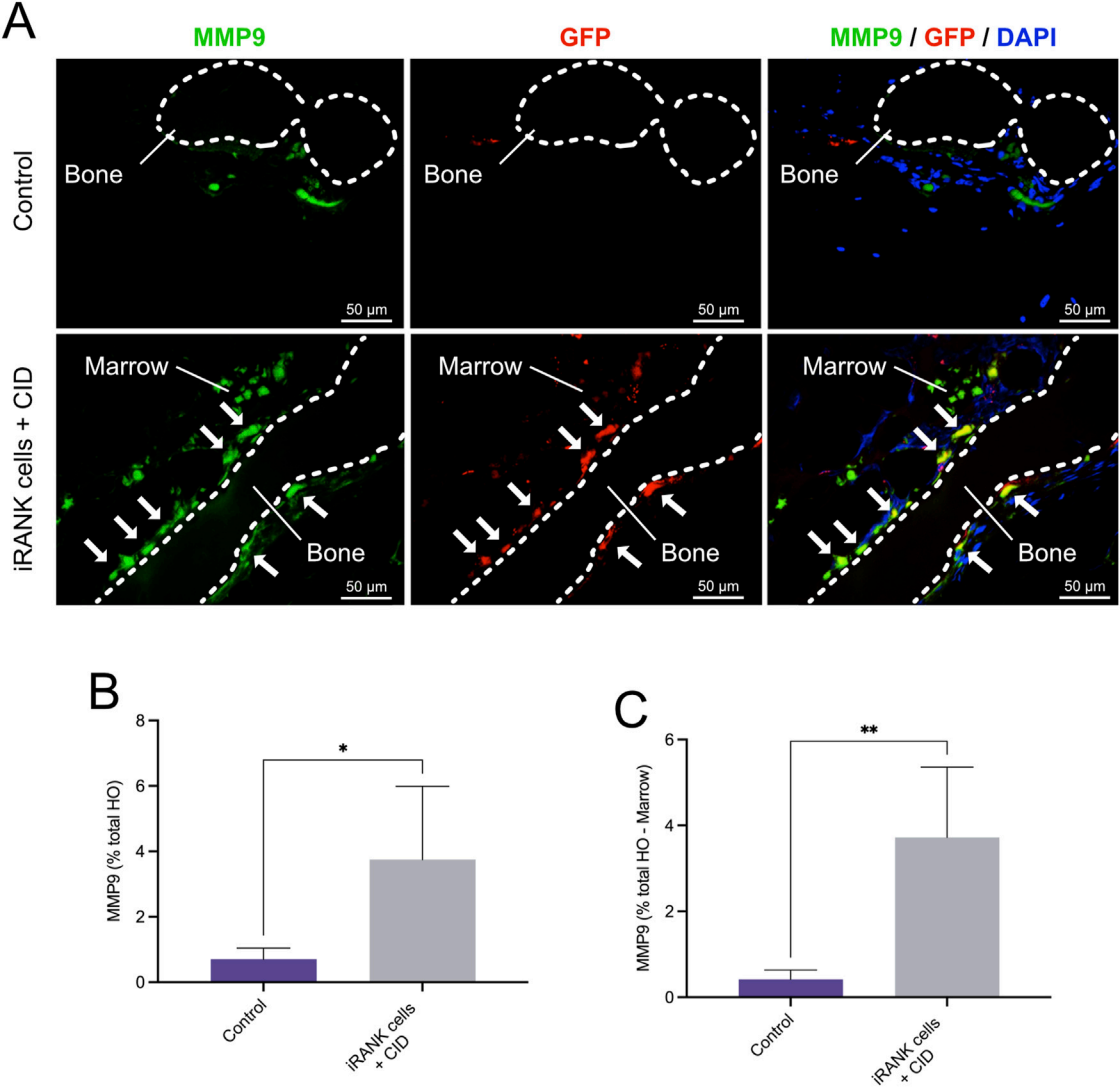
Immunofluorescence staining of MMP9^+^ osteoclasts in HO formations. **(A)** Double immunofluorescence staining showing numerous MMP9^+^GFP^+^ multinucleated OCs lining bone surfaces (B = Bone) with some MMP9^+^GFP^+^ mononuclear cells in the marrow spaces (M = Marrow) in the iRANK treatment group, while only a few MMP9^+^GFP^−^ endogenous osteoclasts were found in the control group. **(B)** MMP9^+^ cells were quantified as percentage of signal intensity per total HO area. **(C)** Results remained statistically significant after subtracting the marrow-like area in which predominantly MMP9^+^ mononuclear cells were found, as assessed by Student’s t-test (n = 3, *p < 0.05, **p < 0.01).

To further confirm this and assess OC activity, we performed double immunofluorescence staining for MMP9, GFP and nuclei on the same tissue sections (Figure 8A). Multiple MMP9^+^GFP^+^ multinucleated cells lining the bone surfaces were observed in the HO formations treated with iRANK cells and CID (white arrows), while only a few MMP9^+^GFP^−^ endogenous multinucleated cells were found in the control group (Figure 8A), consistent with results observed in Figures 7E, F. Two weak signals for GFP were detected in the control group, which were interpreted as unspecific background staining. Only a small number of mononuclear MMP9^+^ cells were observed in the control group (Figure 8A) compared to the HO formations treated with iRANK cells and CID. Most MMP9^+^ cells were located in the adjacent marrow space in both groups.

Overall, significantly more MMP9^+^ cells were observed in the HO formations treated with iRANK cells and CID compared to the control group as measured by percentage of signal intensity per total HO area (Figure 8B). Consistent with the findings, results remained statistically significant after subtracting the marrow-like area in which predominantly MMP9^+^ mononuclear cells were found (Figure 8C).

## 4 Discussion

Surgical removal is currently the only effective option for treating symptomatic HOs. However, complications and limitations are associated with surgical removal. Recurrence of HO formation following surgical removal is frequently observed and may increase in size (Xu et al., 2018; Kazezian and Bull, 2021). Furthermore, surgical intervention increases the risk of perioperative complications such as neurovascular injuries, hemorrhage and wound healing complications. Additionally, resectability of HO formations may be limited based on size, anatomical location, and patient comorbidities (Agarwal et al., 2017).

As pharmacological treatments have demonstrated minimal efficacy (Łęgosz et al., 2018), new therapies to treat existing HO formations are needed. In this study, we investigated the use of engineered OCs to resorb ectopic calcification *in vitro* and treat HO in an *in vivo* mouse model. This cell-based therapy approach could potentially allow for the treatment of inoperable patients by providing a non-surgical alternative, reducing the risks associated with surgery, such as neurovascular injuries and hemorrhage. The targeted injection of engineered myeloid precursors, like iRANK cells, followed by their controlled activation, could facilitate HO resorption and help minimize recurrence compared to traditional surgical removal. Additionally, the therapy is less invasive, allowing for more precise and controlled interventions, ultimately improving patient outcomes while reducing the likelihood of complications and the need for repeated surgeries.

Among potential HO treatments, cell therapy is a compelling strategy. OCs are promising candidates as they normally play a role in bone resorption (Edwards and Weivoda, 2012). In 2021, Jin W et al. developed tetracycline (TC)-modified OCs (TC-OCs) as a potential treatment for HO. They showed that the migration, adherence to bone surface, and bone resorption capacity were increased in TC-OCs when compared to natural OCs *in vitro*. Furthermore, they delivered TC-OCs to HOs using animal models and showed that the HO formations were resorbed in the TC-OC cell-delivery group (Jin et al., 2021). This study was the first to provide proof of principle that OCs might be useful as a HO treatment. On the other hand, as pointed out by Jin et al. (2021), TC coating of OCs was required for effective resorption, nevertheless, are not easily removed by conventional methods (Li et al., 2011). Thus, TC-induced off target disturbances to the bone milieu were recognized as a major limitation of TC-OCs for use in cell therapy (Cheng et al., 2012). Furthermore, TC-OCs would be regulated by endogenous OC inducers and inhibitors, further limiting their clinical application, especially in *in vivo* conditions where OPG levels are high.

To address these limitations, we developed engineered OCs by introducing a conditional RANK signaling system in myeloid precursors. The conditional differentiation of monocytic precursors into OCs relies on the principle of protein interactions through enforced oligomerization of the RANK intracellular signaling domain via a CID (Rementer et al., 2013). The CID system allows for abundant expression of RANK intracellular domain and therefore RANK signaling in OC precursors, is independent of natural OC inducers and inhibitors such as RANKL and OPG, respectively, and can be controlled by discontinuation of CID treatment (Rementer et al., 2013). Importantly, engineered OCs generated from myeloid precursors using the CID system displayed more robust multinucleation and resorption properties than those generated via RANKL alone (Rementer et al., 2013).

In this study, we showed that iRANK cells were able to adhere and differentiate not only on murine calvarial bone, but also on human and mouse HO *in vitro*. While one group of iRANK cells on murine HO nodules showed a clear regression in BV when treated with the CID and the second group steady values, one group shows a clear increase in BV (Figure 4B). When comparing this group to group one in Figure 4A, which were not treated with the CID, similar values in BV can be seen. Even though no irregularities were noted while performing the experiments in this group, technical errors or issues during seeding of iRANK cells on the nodule could explain the lack of regression in bone volume. Alternatively, variability in cellular response could also explain the observation.

In addition to *in vitro* studies, we developed a HO model in nude mice along with iRANK cell delivery and CID-induced OC differentiation methods *in vivo*. Engineered OCs secreted abundant levels of phosphorylated OPN, which likely contributes to their excellent ability to adhere and differentiate on ectopic bone surfaces, as well as inhibit passive mineralization. Phosphorylated OPN may act similarly to the TC used in a previous study to improve adhesion of OCs to ectopic mineral (Jin et al., 2021). Taking µCT, histological, and immunofluorescence analyses together, we showed that the locally delivered iRANK cells were retained at the HO site *in vivo* and differentiated into OCs (TRAP^+^MMP9^+^GFP^+^ multinucleated cells) which resorbed and reduced HOs. These data provide proof-of-concept for restoring bone resorptive functions in HO using engineered OCs and mark a step towards the usage of engineered OCs as a study tool and cell therapy approach to treat symptomatic HOs.

These are the first studies to examine the ability of iRANK cells to differentiate into OCs and resorb HO following treatment with CID *in vivo*. Engineered OC were multinucleated and expressed GFP, in addition to typical OC markers, TRAP and MMP9. Furthermore, they were often localized at the bone surface which in combination with MMP9 expression suggests active resorption. Consistent with this, quantitation showed significantly more MMP9^+^ cells and decreased HO volumes based on µCT scans in HO formations treated with iRANK and CID compared to the control group. Thus, these studies confirm that engineered OCs were formed following iRANK cell delivery and CID treatment and facilitated HO resorption.

Engineered OCs may be useful for treating other pathologies in addition to HO, especially those involving OC deficiency and ectopic calcifications. We previously utilized the iRANK OCs in a medication-related osteonecrosis of the jaw (MRONJ) mouse model (Buranaphatthana et al., 2021). MRONJ is a serious side effect of antiresorptive drugs such as bisphosphonates and denosumab. These drugs lead to OC deficiency by inhibiting OC bone resorptive function (Martínez-Reina et al., 2022), thereby leading to accumulation of necrotic bone and impaired wound healing at the jaw in susceptible patients (Ruggiero et al., 2014). By delivering iRANK cells and CID, bone resorptive function was restored, and necrotic bone area was significantly decreased *in vivo* (Buranaphatthana et al., 2021). This loss-and-gain-of-OC-function study not only supports that OC inhibition play a role in accumulation of necrotic bone, but also introduces OC cell therapy as a potential MRONJ treatment. Thus, our studies, combined with those of Jin et al. (2021) suggest that engineered OC delivery may be useful to treat diseases requiring resorption of unwanted bone, including HO, MRONJ and ectopic calcification.

Several limitations to our study exist. As allogenic and not autologous iRANK cells were used, a nude mouse model of HO was utilized to avoid unfavorable host responses due to cell delivery. While we showed that HOs formed in nude mice within a similar time course, size, and morphology to those in wild-type mice, the lack of a full immune response may have altered the HO formations and their ability to be resorbed. Second, iRANK cells constitute myeloid precursors that can differentiate toward macrophage lineages in the absence of CID (unpublished data). This could exacerbate inflammation around HO formations, as described previously (Buranaphatthana et al., 2021). Furthermore, current iRANK cells do not represent a viable cellular treatment option for patients. Generation of human-engineered OCs that are not derived from malignant cells will be needed to utilize this approach in HO patients in the future (Blümke et al., 2023; Blümke et al., 2024).

As part of this study, local delivery of iRANK cells onto HO formations was done using a collagen hydrogel. While luciferase, blood studies, and bone analyses did not show migration of cells beyond the injection side, this possibility cannot be ruled out. One possibility to restrict iRANK differentiation to the vicinity of the HO formations would consist of using a slow-release vehicle to deliver CID directly to the HO site, rather than systemic delivery. Another approach is to deliver purified engineered OCs that have been differentiated *in vitro*. In either case, precision targeting of the CID and/or iRANK cells will be required to achieve efficient HO resorption. Finally, AP20187, the CID used in this study, has been reported to cause transient thrombocytopenia in a dog model (Okazuka et al., 2011). Although AP20187 was widely used during the research period, alternative CIDs like AP1903 have demonstrated greater safety and tolerability in human patients (Iuliucci et al., 2001).

Future steps for this novel therapy include developing human iRANK cells to minimize immune responses and improve safety. Since the resorption of HO depends on the delivery location of the cells, there is a risk of healthy bone being resorbed if iRANK cells come into contact with it. Therefore, optimizing delivery methods for the precise placement of iRANK cells around the HO will be essential for effective clinical applications. Furthermore, concerns regarding side effects of the CID treatment, malignant conversion of human iRANK cells and the fate of iRANK cells following the completion of treatment need to be addressed.

## 5 Conclusion

In conclusion, our study demonstrated that engineered myeloid precursors can differentiate into OCs and effectively resorb HO in mice. This approach shows promise as a potential nonsurgical therapy for HO, offering a new avenue for treatment that addresses the limitations and complications associated with current surgical options.

## Data Availability

The original contributions presented in the study are included in the article/Supplementary Material, further inquiries can be directed to the corresponding author.
