# Supplementary material for: Engineered myeloid precursors differentiate into osteoclasts and resorb heterotopic ossification in mice
Source: Front Bioeng Biotechnol. 2024 Nov 22;12:1491962. doi: 10.3389/fbioe.2024.1491962 (PMC11620886; doi:10.3389/fbioe.2024.1491962)
Supplement: Supplementary file 1 [file DataSheet1.docx]

Supplementary Material

# Supplementary Materials and Methods

## Preparation of Cell Delivery Vehicles for *In Vivo* Delivery

Collagen and Basement Membrane Extract (BME) were tested as cell delivery vehicles. To formulate the collagen carrier, acid-solubilized collagen was diluted with both 10X PBS and a solution of 10 N sodium hydroxide to give a final concentration of 0.5 mg/mL collagen. The solution was kept on ice until injection.
To formulate the BME carrier, frozen Cultrex BME (R&D Systems, Minneapolis, MN) was thawed overnight and diluted with PBS to a final concentration of 0.5 mg/mL BME. Cells were added immediately to both vehicles before injection.

## Lentiviral production and transduction of luciferase gene into iRANK cells

The packaging plasmids pSL3 (vesicular stomatitis virus G envelope), pSL4 (HIV-1 gag/pol packing genes) and pSL5 (rev gene required for HIV-1 envelope protein expression) were a gift from the Murry Lab (University of Washington, Seattle, WA). The transfer plasmid pLEL (luciferase gene) was a gift from the Pub Lab (University of Washington, Seattle, WA). The lentiviral vector was packaged in HEK293T cells as previously described (Rementer et al., 2013) with the following modifications: Briefly, a total of 5 × 10^6^ of HEK293T cells were seeded in 10-cm dishes treated with poly-D-lysine 24 hours prior to transfection and the culture media was changed just before transfection. A total 20 μg plasmid DNA (7.5 μg pLEL, 2.5 μg pSL3, 6.7 μg pSL4 and 3.3 μg pSL5) was used for the transfection of one dish. The plasmids were added to 4 mL of Opti- MEM (Thermo Fisher Scientific, Waltham, MA). This was combined with 4 mL of Opti-MEM to which 120 μL of Lipofectamine 2000 has been added (Thermo Fisher Scientific, Waltham, MA). The solution was added to the cultures and the media replaced after 14-16 hours. The media containing virus was collected after another 48 hours and filtered through a 0.45-μm filter. Various volumes of the media containing virus was applied to the target cells, RAW264.7 iRANK cells for overnight incubation. Transduced RAW264.7 iRANK cells were allowed to expand before use. Luciferase expression was confirmed and quantified via luminometer.

## *In Vivo* Bioluminescence imaging for visualization of iRANK cells following *In Vivo* Delivery

For *in vivo* imaging, D-luciferin Firefly potassium salt (PerkinElmer, Waltham, MA) was prepared at a 15 mg/ml stock in PBS and filtered through a 0.22-μm filter before use. Mice were injected intraperitoneally with 10 μL of stock solution per gram of body weight 10 to 15 minutes before imaging. Mice were then anesthetized by inhalation of isoflurane and were positioned at the nose cones in the Xenogen IVIS imaging system. Images were taken by using the IVIS-200 Imaging System (Xenogen Corporation, Alameda, CA).

## iRANK Cell Differentiation Studies

iRANK cells in media were mixed with delivery vehicles and seeded into wells where they formed gels. Media containing the CID was added to each well to induce osteoclast formation as described above. After 4-7 days, the cells were fixed, and TRAP staining performed. The quantitation of TRAP^+^ OCs shows that CID-induced OCs were observed in all groups indicating that none of the vehicles hindered CID-induced osteoclastogenesis.

# Supplementary Figures

**Supplementary Figure S1.** **Development of cell delivery vehicles for *in vivo* iRANK cell delivery**. (A) Wild-type C57BL/6 mice (n = 2 per group) received bilateral injections of cells and vehicles for a total of 4 injection sites per condition. Mice were imaged 5 times (day 2, 3, 4, 5 and 8) following luciferin injection. Only images from day 8 are shown in panel A. (B) The radiance unit as the number of photons per second that leave a square centimeter of tissue and radiate into a solid angle of one steradian (sr) (p/s/cm2/sr) was used as the quantitative data of the bioluminescence imaging. The radiance was increased overtime in all groups indicating that iRANK cells were retained and proliferated at the delivery site. (* p < 0.05 compared collagen to no gel group; # p < 0.05 compared collagen to BME group) (C) iRANK cells were treated with CID in the presence of different vehicles to determine if they interfere with OC differentiation *in vitro*. The quantitation of TRAP^+^ multinucleated OCs per well showed that CID-induced OCs were observed in all groups indicating that none of the vehicles hindered CID-induced osteoclastogenesis.
**Supplementary Figure S2.** **Development of HO nodules in nude mice.** Prior to initiating cell delivery studies, we performed a pilot study to confirm that the absence of lymphocytes in nude mice does not affect HO formation. For this pilot study, nude mice were anesthetized and a Matrigel carrier impregnated with 2.5 µg BMP-2 was implanted in the mid-belly of the gastrocnemius muscle. HO formation was evaluated by µCT at 10, 17, and 28 days post-implantation and the volume of HO formations and the percent mineralization was calculated. Serial µCT images show that mineralizing ectopic bone (orange) was located in the mid-belly of the gastrocnemius muscle group, posterior of the tibia (t) and medial to the fibula (f). At Day 28, the average bone formation volume was 1 mm^3^ and the percent mineralization (BV/TV) was 24.7%.


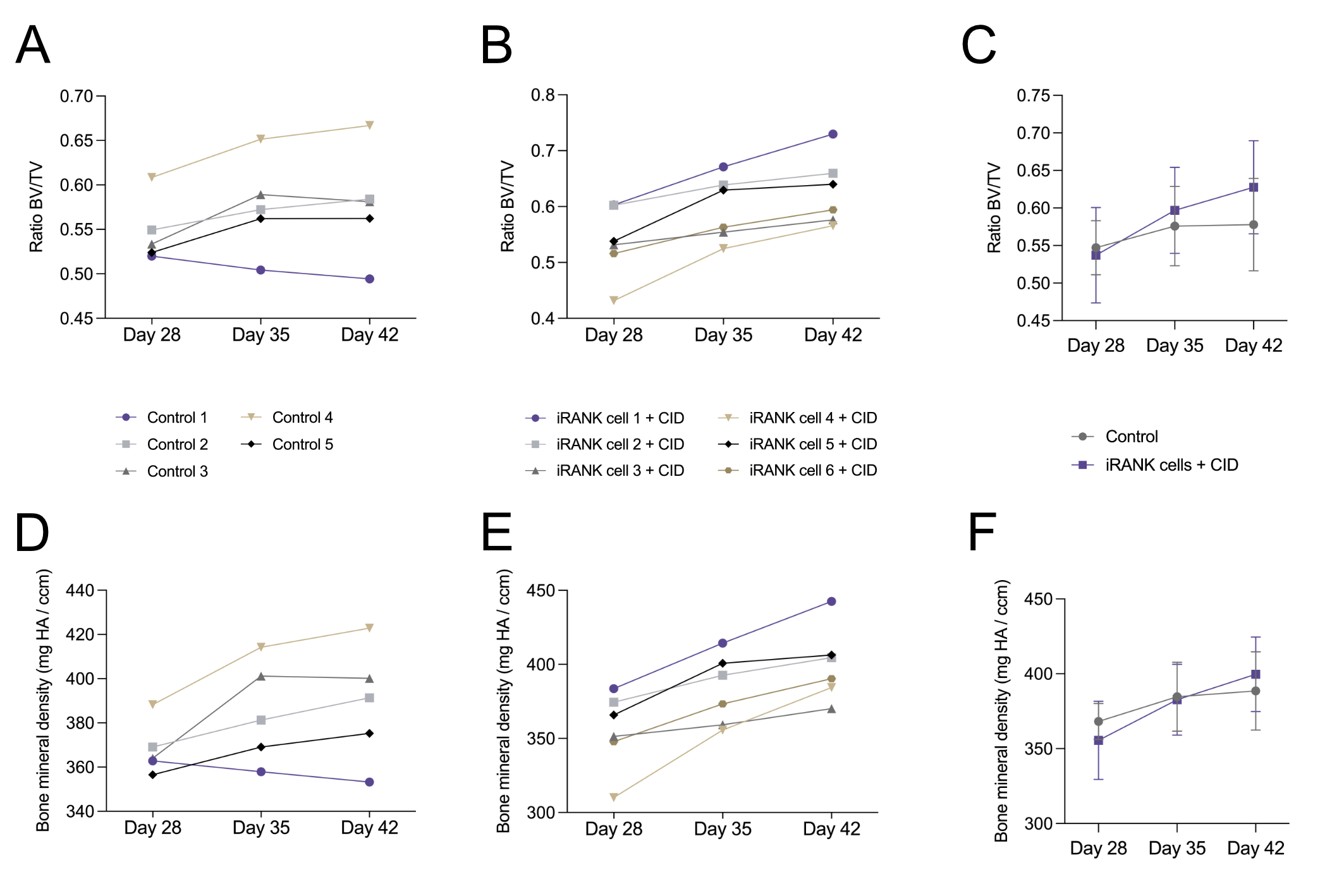
 **Supplementary Figure S3. BV/TV ratio and BMD measurements of *in vivo* HO formations (Supplementary data to Fig. 6).** (A-C) The ratio of bone volume (BV) to total tissue volume (TV) is shown. No statistically significant differences were observed between the control and treatment groups. (C) A slight, although statistically non-significant, increase in the BV/TV ratio was noted in the treatment group, which may be attributed to a greater decrease in TV compared to BV, as shown in Fig. 6. (D-F) Bone mineral density (BMD) also did not differ significantly between the control and treatment groups.

# References

Rementer, C. W., Wu, M., Buranaphatthana, W., Yang, H. Y. L., Scatena, M., and Giachelli, C. M. (2013). An inducible, ligand-independent receptor activator of NF-κB gene to control osteoclast differentiation from monocytic precursors. *PLoS One* 8. doi: 10.1371/JOURNAL.PONE.0084465
